# Quantifiable features of a tidal breathing phenotype in dogs with severe bronchomalacia diagnosed by bronchoscopy

**DOI:** 10.1080/01652176.2023.2252518

**Published:** 2023-09-04

**Authors:** Chung-Hui Lin, Lynelle R. Johnson, Wei-Tao Chang, Pei-Ying Lo, Hui-Wen Chen, Huey-Dong Wu

**Affiliations:** a National Taiwan University Veterinary Hospital, National Taiwan University, Taipei, Taiwan; b Graduate Institute of Veterinary Clinical Sciences, School of Veterinary Medicine, National Taiwan University, Taipei, Taiwan; c TACS-Alliance Research Center, Taipei, Taiwan; d Department of Medicine and Epidemiology, The University of California School of Veterinary Medicine, Davis, CA, USA; e Department of Veterinary Medicine, National Taiwan University, Taipei, Taiwan; f Section of Respiratory Therapy, Department of Integrated Diagnostics and Therapeutics, National Taiwan University Hospital, National Taiwan University, Taipei, Taiwan

**Keywords:** Bronchomalacia, bronchoscopy, dogs, functional phenotype, lower airway obstruction, pulmonary function test, spirometry, tidal breathing

## Abstract

Dynamic lower airway obstruction is the primary component of canine bronchomalacia, but the ventilatory function remains underinvestigated. This prospective study analyzed tidal breathing characteristics in 28 dogs, comprising 14 with severe bronchomalacia diagnosed by bronchoscopy versus 14 without respiratory disease. Spirometry was conducted in all dogs. Bronchoscopy with bronchoalveolar lavage or brush under anesthesia was performed in 14 dogs with cough and expiratory effort. Severe bronchomalacia was defined by the severity of collapse and total number of bronchi affected. Ventilatory characteristics were compared between groups. Results revealed that dogs with severe bronchomalacia had lower minute volume (218 vs 338 mL/kg, *p* = .039) and greater expiratory-to-inspiratory time ratio (1.55 vs 1.35, *p* = .01) compared to control dogs. The tidal breathing pattern of dogs with bronchomalacia was different from that of normal dogs, and the pattern differed from the concave or flat expiratory curves typical of lower airway obstruction. Compared to control dogs, dogs with severe bronchomalacia had a significantly prolonged low-flow expiratory phase (*p* < .001) on the flow-time plot and a more exponential shape of the expiratory curve (*p* < .001) on the volume-time plot. Flow-time index ExpLF/Te (>0.14) and volume-time index Vt-AUCexp (≤31%) had a high ROC-AUC (1.00, 95% confidence interval 0.88 to 1.00) in predicting severe bronchomalacia. In conclusion, the tidal breathing pattern identified here indicates abnormal and complicated ventilatory mechanics in dogs with severe bronchomalacia. The role of this pulmonary functional phenotype should be investigated for disease progression and therapeutic monitoring in canine bronchomalacia.

## Introduction

Canine bronchomalacia refers to the excessive collapsibility of the principal, lobar, segmental, subsegmental, or other smaller bronchi (Johnson and Pollard 2010; Adamama-Moraitou et al. 2012; Singh et al. 2012; Bottero et al. 2013; Johnson et al. 2015; Reinero and Masseau 2021). The number of bronchi involved and the extent of collapse in malacic segments in each affected dog can be extremely variable, and currently there is no universally accepted consensus for the classification (Johnson and Pollard 2010; Adamama-Moraitou et al. 2012; Singh et al. 2012; Bottero et al. 2013). Although concurrent tracheal and bronchial collapse is commonly present and termed as tracheobronchomalacia in the veterinary literature, bronchomalacia can be an isolated finding in the absence of tracheal collapse (Johnson and Pollard 2010; Bottero et al. 2013). In contrast to tracheal collapse, a disorder of small-breed dogs, bronchomalacia is also commonly found in medium- to large-breed dogs (Johnson and Pollard 2010; Adamama-Moraitou et al. 2012; Bottero et al. 2013). To date, the exact etiology of canine bronchomalacia remains unknown, although certain factors such as persistent inflammation, cartilage degeneration, and genetic/breed predisposition have been suspected (Johnson and Pollard 2010; Adamama-Moraitou et al. 2012; Bottero et al. 2013; Reinero and Masseau 2021). Cough is usually the primary respiratory complaint, followed by sporadically reported respiratory distress or increased expiratory effort in some dogs (Johnson and Pollard 2010; Adamama-Moraitou et al. 2012; Hara et al. 2019). There has not yet been a proven effective treatment specific for canine bronchomalacia, leading to the importance of maintaining an ideal body weight and recognizing all comorbidities as available options for current management. Thoracic radiographs generally underestimate the presence of bronchomalacia, and the incidence of bronchomalacia among dogs with respiratory disease undergoing bronchoscopy was estimated to be 50% (Johnson and Pollard 2010). Bronchoscopy is the current gold standard method to diagnose bronchomalacia, to document the severity, and to identify concurrent etiologies (Johnson and Pollard 2010; Adamama-Moraitou et al. 2012; Bottero et al. 2013; Johnson et al. 2015; Wallis et al. 2019). Computed tomography using a 320-slice scanner with sedation or by paired breath-hold method under anesthesia has also been utilized for diagnosis in two recent studies (Hara et al. 2019; Levy et al. 2022).

Dynamic lower airway obstruction is the primary pathophysiologic component of canine bronchomalacia. Pulmonary function testing is of great importance in understanding the type and severity of obstructive lower airway or lung disease in human clinical medicine, and lower respiratory tract obstruction is conventionally described as a concave or flat expiratory curve on tidal or forced breathing flow-volume loops (Hyatt 1975; Amis and Kurpershoek 1986; Padrid et al. 1990; McKiernan et al. 1993; Weiner et al. 2003; Varga et al. 2016). Tidal breathing analysis by spirometry has been applied in dogs as a pulmonary function assessment in various diseases, including chronic bronchitis, tracheal collapse, and upper airway obstruction (Amis and Kurpershoek 1986; Padrid et al. 1990; Rozanski and Hoffman 1999; Pardali et al. 2010; Benavides et al. 2019). However, ventilatory function has not yet been fully investigated in dogs with bronchomalacia.

The purposes of this study were to analyze the tidal breathing characteristics in dogs with expiratory effort due to bronchomalacia, and to evaluate quantifiable indices that could assist in identifying severe bronchomalacia.

## Materials and methods

### Animals

**Figure 1.**
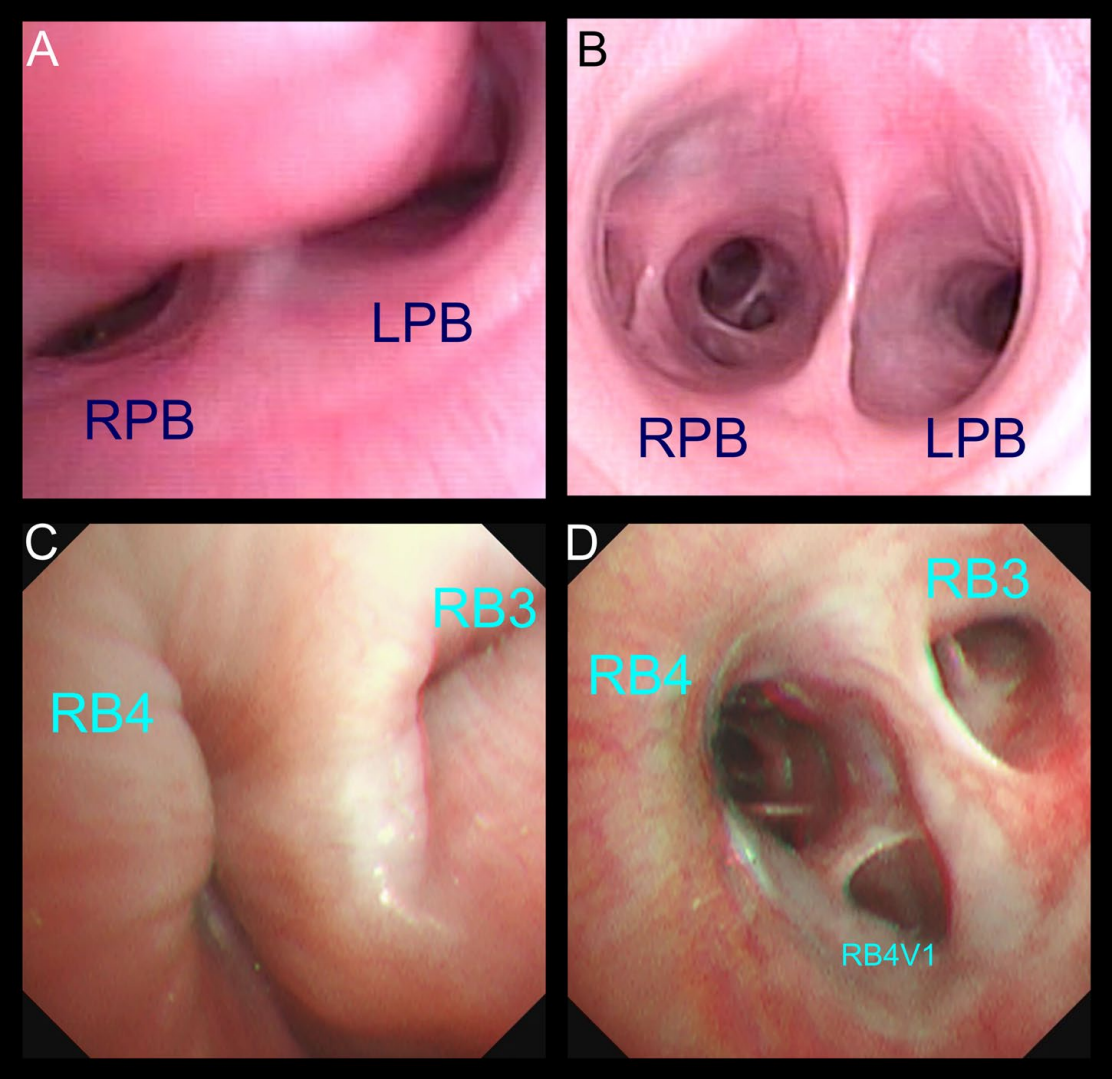
The definitive diagnosis of canine bronchomalacia was made by bronchoscopy in this study. (A) Bronchoscopic view showing severe collapse of the left and right principal bronchus (LPB and RPB). (B) Bronchoscopic image at the same level of the carina from a dog without bronchomalacia. (C, D) Bronchoscopic images demonstrating the dynamic collapse of the lobar bronchus of the accessory lobe (RB3) and the right caudal lobe (RB4) in a dog with severe bronchomalacia.

Twenty-eight dogs that cooperated for spirometric recordings were prospectively included in this study. Fourteen of the 28 were clinical canine patients presenting for cough and showing variable expiratory effort, and a definitive diagnosis of canine bronchomalacia was made by bronchoscopy (Figure 1). Dogs were excluded from the study if: (i) the overall clinical evaluation was not able to clarify the underlying etiology of presenting signs; (ii) a high anesthetic risk was determined and bronchoscopy could not be performed; (iii) the final diagnosis responsible for the clinical signs was other than severe bronchomalacia (e.g. if concurrent alveolar, interstitial, or pleural space disease was identified by thoracic radiography, ultrasonography, or other clinical examination). Control dogs (*n* = 14) were recruited from pet owners or university staff and were free of respiratory signs in the preceding six months. All control dogs were required to have a normal physical examination by an attending veterinarian, as well as normal blood work and thoracic radiographs. Dogs were excluded from the control group if there were any of the following findings: (i) adventitious respiratory sounds detected on physical examination by a senior veterinarian; (ii) spirometric waveforms dissimilar to those reported for normal dogs in previous literature (Amis and Kurpershoek 1986); (iii) acute respiratory disease or infectious episode within the past two months. The study was approved by the Institutional Animal Care and Use Committee (IACUC) of National Taiwan University (Approval No: NTU104-EL-00010 and NTU107-EL-00163).

### Bronchoscopy

Bronchoscopy was performed under general anesthesia using an anesthetic plan designed specifically for each dog based on the patient’s overall health status. A 3.1 mm (BF-XP290, Olympus) or 5.3 mm (VISERA PEF-V, Olympus) flexible video endoscope was used. In brief, bronchoscopic examination was performed in sternal recumbency, and the canine bronchial map by Amis and McKiernan was used for identification of lower airways (Amis and McKiernan 1986). The entire evaluation and the sampling sites/methods were based on the individual patient’s condition.

The luminal size of the left principal bronchus (LPB), the right principal bronchus (RPB), and lobar bronchi of left cranial lobe (LB1), left caudal lobe (LB2), right cranial lobe (RB1), right middle lobe (RB2), accessory lobe (RB3), and right caudal lobe (RB4) was assessed, as well as all segmental and subsegmental bronchi that could be evaluated. To describe the extent and the distribution of bronchial collapse in this study, the criteria were as follows. An airway was considered normal if the bronchial opening was round to ovoid with the change of luminal size during respiration of less than 25%. Reduction of luminal size was classified into 4 levels: between 25–50%, approximately 50%, between 51–75%, or > 75%. Dynamic bronchial collapse was documented when the maximal reduction of luminal size during respiration was greater than 25%. The overall severity of bronchomalacia for each case was graded as absent, mild, moderate, or severe, taking into account both the worst level of collapse identified and the total number of airways affected (Table 1) (Johnson and Pollard 2010; Singh et al. 2012; Bottero et al. 2013). Inflammatory airway disease was defined as increased inflammatory cell percentage in the cytology of bronchoalveolar lavage (BAL) (neutrophils, eosinophils, or lymphocytes > 8%) or bronchial brush (if neutrophils, eosinophils, or lymphocytes > 2%) specimens after excluding other possible etiologies such as infection by bacterial culture (Hawkins et al. 2006; Johnson and Pollard 2010; Zhu et al. 2015; Johnson and Vernau 2019).

**Table 1.** The overall severity of bronchomalacia was based on the degree of collapse and the total number of airways affected.

Grade of severity	Degree of collapse	Total number of airways with collapse ≥ 50%
Mild	Affected airways between 25 to 50% or up to 50% collapse	≤ 2 of eight sites affected
Moderate	The overall degree of collapse between mild and severe grade	The number of airways affected between mild and severe grade
Severe	≥ 3 bronchi reach 51–75% collapse **OR** At least 1 bronchus >75% collapse	≥ 3 of eight sites plus segmental bronchi

*Notes:* Total possible airways affected included eight sites: LPB, RPB, LB1, LB2, RB1, RB2, RB3, RB4. When segmental bronchi were affected, this was considered as a ninth site and the required criterion for the highest severity.

Abbreviations: LPB: the left principal bronchus; RPB: the right principal bronchus; LB1: lobar bronchus of left cranial lobe; LB2: lobar bronchus of left caudal lobe; RB1: lobar bronchus of right cranial lobe; RB2: lobar bronchus of right middle lobe; RB3: lobar bronchus of accessory lobe; RB4: lobar bronchus of right caudal lobe.

### Spirometric recording

**Figure 2.**
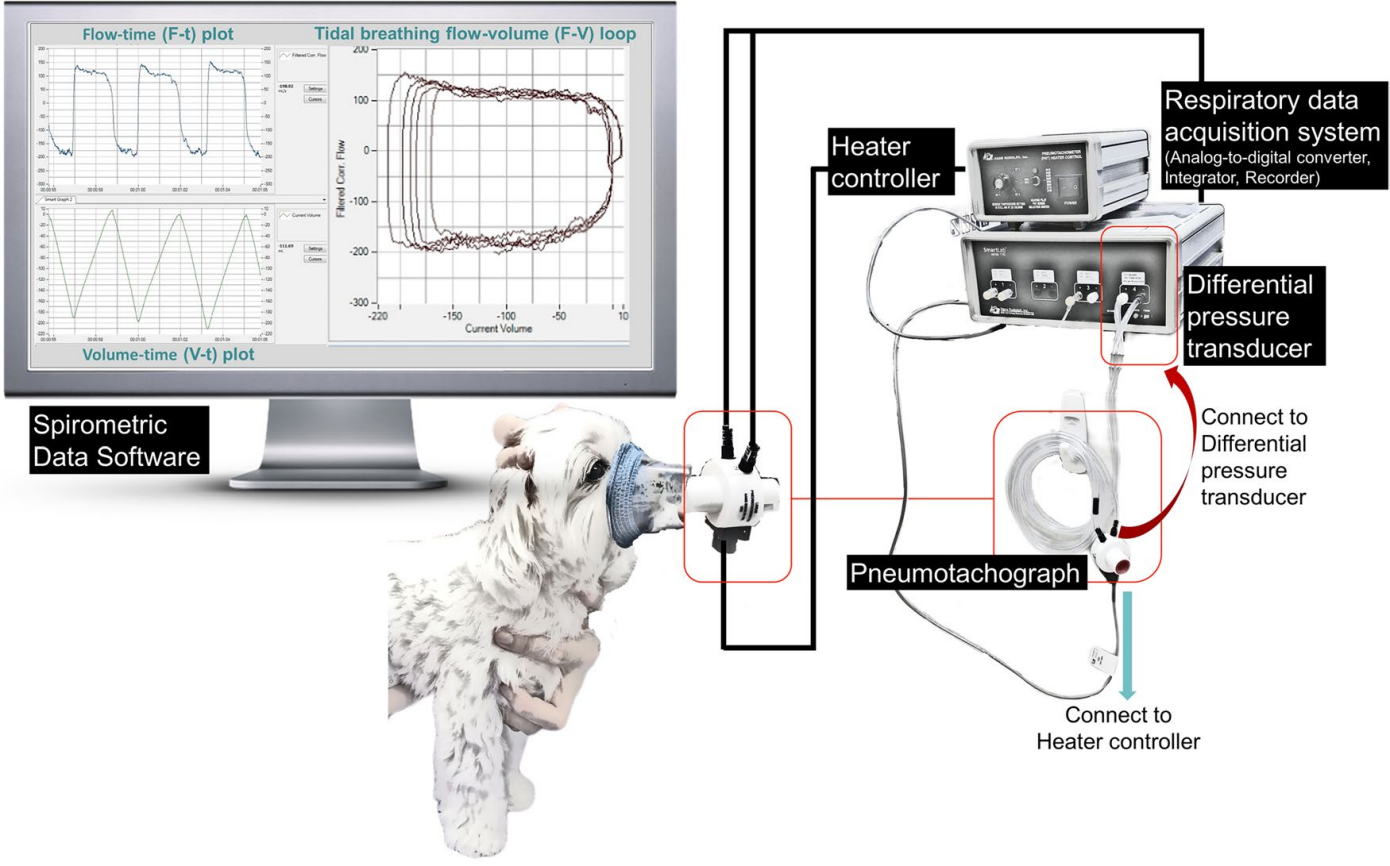
Diagram of the set-up used to measure tidal breathing signals in dogs.

For recording of spirometry (Figure 2), dogs were awake and gently restrained in a standing, sitting, or sternal position in a quiet room. Before the recording, dogs were acclimated to the environment, the personnel, and a snugly-fitted anesthetic facemask with rubber gasket for several minutes. In the process of recording, the head of the dog was carefully maintained in a neutral position without compressing the airway, while simultaneously monitoring the dog’s breathing status to detect non-tidal breathing events (e.g. coughing, licking, swallowing). The facemask was attached to a pneumotachograph, which was chosen based on the dog’s size and the airflow range to be measured (e.g. a pneumotachograph with flow range of 0–35 or 0–160 liter per minute). Seven of the 28 dogs were recorded using a disposable pneumotach (PN 281637 and PN 260177, Hamilton) with a research pneumotach system (RSS100, Hans Rudolph Inc., Shawnee, Kansas, USA). The spirometric system was updated during the study period, and a heated linear pneumotachograph (Model 3500B and 3700B, Hans Rudolph Inc., Shawnee, Kansas, USA) was used to improve the signal quality. The pneumotachograph was calibrated based on the manufacturer’s instruction and validated using a volume calibration syringe (5540 or 5570, Hans Rudolph Inc., Shawnee, Kansas, USA) to certify accuracy within 2% prior to each data collection. A differential pressure transducer (1140 A, Hans Rudolph Inc., Shawnee, Kansas, USA) and a respiratory data acquisition system (SmartLab™, Hans Rudolph Inc., Shawnee, Kansas, USA) were connected to the pneumotachograph. The signals were digitized and analyzed using commercial software (Insight™ Software, Hans Rudolph Inc., Shawnee, Kansas, USA) with a sampling rate of 200 Hz. Flow signals were integrated to generate inhaled and exhaled volumes. Based on clinical judgment of the dog’s stress or anxiety level, the facemask was intermittently removed from the dog’s muzzle to restore stable and regular breathing (DeVanna et al. 2014). The breathing status of the dog was monitored by both direct observation and visual inspection of the real-time graphical display. The recording was considered to be complete when a repeatable breathing pattern (consistent shape of waveforms with stable volume) was recognized on the real-time graphic plots and at least 5–10 representative breaths were obtained (Amis and Kurpershoek 1986). Data were interpreted by four investigators in this study and the same two investigators (C-H Lin and P-Y Lo) examined each set of spirometric data.

### Quantitative assessment of tidal breathing signals

Basic ventilatory parameters were calculated from breaths free of artifacts and were reported by the software with a breath-by-breath analysis, including respiratory rate (RR, cycles/min), tidal volume per kg body weight (TV/BW, mL/kg), minute volume per kg BW (MV/BW, mL/kg), inspiratory and expiratory time (Ti and Te, second), peak inspiratory and expiratory flow per kg BW (PIF and PEF, mL/s/kg).

The shape of tidal breathing flow-volume (F-V) loops was assessed as previously reported (Amis and Kurpershoek 1986; Pardali et al. 2010; Benavides et al. 2019). Expiratory or inspiratory flow at end TV plus 50% and 25% TV (EF50, IF50, EF25, IF25) was calculated by the software. Loop indices for delineating overall loop shape were calculated and analyzed from 5 to 10 representative breaths, including PEF/PIF, PEF/EF50, PEF/EF25, EF50/EF25, PIF/IF50, PIF/IF25, IF50/IF25, EF75/IF75, EF50/IF50, and EF25/IF25.

The flow-time (F-t) and volume-time (V-t) plots were simultaneously recorded by the system software, and signal tracings of 5 to 10 representative breaths were inspected for a repeated qualitative pattern. The parameters for delineating the graphic characteristics of F-t and V-t plots were modified from previous studies (McKiernan et al. 1993; Clarke et al. 1994; Burnheim et al. 2016).

### Statistical analysis

Data with continuous variables were expressed as median and range. Commercial statistical software (SPSS 25; IBM Corporation, Armonk, NY; MedCalc, version 19.6, MedCalc Software, Ostend, Belgium) was used for all analyses, and the level of significance was set at *p* <.05. Age, body weight, body condition score, and all respiratory data were compared between groups using the Mann Whitney U test. Comparison of the spirometric results between dogs with severe bronchomalacia and control dogs was repeated after excluding the data of 7 dogs recorded by the pneumotach prior to the system update. The sensitivity and specificity of spirometric parameters for predicting severe bronchomalacia were evaluated by receiver operating characteristic curves.

## Results

### Study population

Breeds of dogs with bronchomalacia in the study consisted of seven Miniature and Toy Poodles, three cross-breeds, and one Cocker Spaniel, Yorkshire Terrier, Chihuahua, and Pomeranian. There were five cross-breeds, three Miniature and Toy Poodles, two Dachshunds, and one Maltese Terrier, West Highland Terrier, Chihuahua, and Miniature Schnauzer in the control group. Eight (six neutered) and nine (six neutered) were females in the bronchomalacia and control group respectively. The median age of all 28 dogs was 11 years (range, 1–16), without statistical difference between dogs with bronchomalacia and control dogs (11 vs 8.3, *p* = .09). The median bodyweight of dogs with bronchomalacia was statistically smaller than control dogs (4.1, range 2.0–11.4 vs 6.5, range 3.3–27.5 kg, *p* = .02), but there was no difference in nine-point body condition score between groups (5 vs 5, *p* = .9). Thoracic radiographs revealed a bronchointerstitial pattern in all 14 dogs with bronchomalacia, and lung hyperinflation was absent in all dogs. There was no clinical suspicion for alveolar, interstitial, pleural space disease, or upper airway obstruction based on signalment, disease history, physical examination (e.g. palpation, hydration and perfusion check, auscultation over the neck region, lung fields, and heart, etc), blood work, and thoracic radiography. Pre-anesthetic blood work was considered unremarkable in all dogs with respiratory disease, therefore bronchoscopy with lower airway specimen collection was performed.

### Bronchoscopic findings

Of 14 dogs diagnosed with severe bronchomalacia, the median number of affected bronchi out of eight sites was 8 (range, 3–8). Principal left or right bronchus was affected in 12/14 dogs, and 8/12 dogs had the most severe collapse (greater than level 3). The left cranial (14/14) and accessory (14/14) lobar bronchi were most commonly affected in these dogs. Segmental bronchi were affected in all 14 dogs, but the severity could not be objectively compared among dogs because not all smaller bronchi could be evaluated in all individuals. Concurrent tracheal collapse was observed in all 14 dogs with bronchomalacia, but the extent of tracheal collapse (grade I: 5, grade II: 6, grade III: 3, grade IV: 0; graded based on the maximal collapse at any point in the trachea) was much less severe than that of bronchomalacia. Eleven out of the 14 dogs had both extrathoracic and intrathoracic tracheal collapse, but 5 out of the 11 had even a mild grade of collapse in either the extrathoracic or intrathoracic segment. Inflammatory airway disease was concurrently identified in 11/14 dogs based on BAL or bronchial brush cytology (3 neutrophilic, 3 lymphocytic, 3 mixed neutrophilic-lymphocytic, and 2 mixed neutrophilic-eosinophilic inflammation). No intracellular bacteria or growth of pathogens was identified in dogs with bronchomalacia.

### Basic ventilatory parameters

Data of the basic ventilatory parameters are shown in Table 2. Dogs with bronchomalacia had a significantly lower MV/BW (218 vs 338 mL/kg, *p* = .039) and greater Te/Ti ratio (1.55 vs 1.35, *p* = .01) compared to control dogs.

**Table 2.** Basic ventilatory parameters (median with range) in dogs with bronchomalacia and control dogs.

Ventilatory parameters	Bronchomalacia (*n* = 14)	Control (*n* = 14)	*p*
RR (cycles/min)	21 (16–42)	26 (17–59)	.19
TV/BW (mL/kg)	9.8 (3.5–20.9)	13.0 (5.4–27.3)	.11
MV/BW (mL/kg)	218 (59–490)	338 (171–879)	.039*
Ti (second)	0.93 (0.62–1.4)	0.98 (0.43–1.56)	.98
Te (second)	1.65 (0.88–2.38)	1.25 (0.58–2.0)	.13
Te/Ti (ratio)	1.55 (1.13–2.76)	1.35 (1.11–1.81)	.011*
PIF/BW (mL/s/kg)	16.5 (3.2–32.2)	15.9 (10.2–54.3)	.48
PEF/BW (mL/s/kg)	28.6 (7.6–43.5)	14.6 (8.7–41.7)	.09

*Note:* Significant differences (*p* < .05) are denoted with boldface.

Abbreviations: RR: respiratory rate; TV/BW: tidal volume per kg body weight; MV/BW: minute volume per kg BW; Ti and Te: inspiratory and expiratory time; PIF/BW and PEF/BW: peak inspiratory and expiratory flow per kg BW.

### Tidal breathing flow-volume (F-V) loop

Conventional features, such as concave or flat expiratory curves on F-V loops, were not observed in the study dogs with severe bronchomalacia. F-V loop indices indicating a concave expiratory curve (elevated PEF/EF50 or PEF/EF25 ratio) were not found in dogs with severe bronchomalacia (Table 3). There were also no differences in PEF/EF50 (1.19 vs 1.21, *p* = .38) and PEF/EF25 (1.71 vs 1.39, *p* = .23) between dogs with bronchomalacia and control dogs. F-V loop indices indicating a flat expiratory curve (reduced PEF/PIF or EF50/IF50) were not noted in these dogs with severe bronchomalacia. On the contrary, PEF/PIF (1.66 vs 0.78, *p* < .001) and EF50/IF50 (1.75 vs 0.81, *p* < .001) were significantly higher in dogs with severe bronchomalacia compared to control dogs. The same conclusion was obtained even after excluding the data of 7 dogs recorded by the system prior to the upgrade.

**Table 3.** Spirometric parameters from flow-volume (F-V) loop, flow-time (F-t) plot, and volume-time (V-t) plot (median with range) in dogs with bronchomalacia and control dogs.

F-V, F-t, and V-t parameters	Bronchomalacia (*n* = 14)	Control (*n* = 14)	*p*
PEF/EF50	1.19 (1.00–1.59)	1.21 (1.06–1.48)	.38
PEF/EF25	1.71 (1.07–3.96)	1.39 (1.19–2.13)	.23
PEF/PIF	1.66 (1.04–2.39)	0.78 (0.59–1.15)	<.001*
EF50/IF50	1.75 (0.96–2.47)	0.81 (0.54–1.20)	<.001*
ExpLF/Te	0.76 (0.57–0.93)	0.05 (0.02–0.14)	<.001*
Vt-AUCexp (%)	19.3 (10.7–31.0)	44.4 (33.9–48.9)	<.001*

*Note:* Significant differences (*p* < .05) are denoted with boldface.

Abbreviations: PIF and PEF: peak inspiratory and expiratory flow; EF50 and IF50: expiratory or inspiratory flow at end TV plus 50% TV; EF25: expiratory flow at end TV plus 25% TV; ExpLF/Te: the ratio of the expiratory low-flow phase to the entire expiratory period; Vt-AUCexp: the proportion of area under expiratory curve on V-t plot.

### Flow-time (F-t) plot

**Figure 3.**
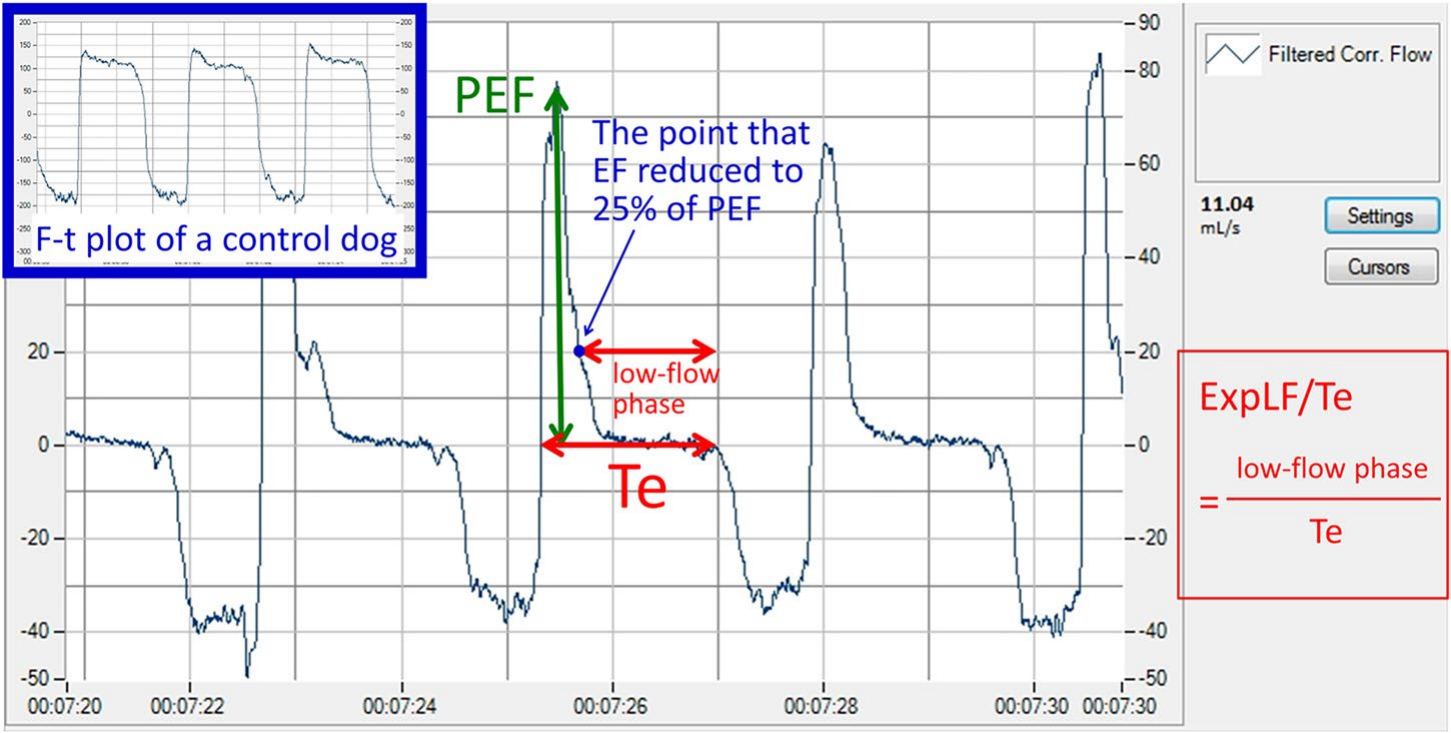
A flow-time (F-t) plot from a dog with severe bronchomalacia, showing a significantly prolonged low-flow expiratory phase. A F-t plot from a control dog was shown on the upper left Corner.

Dogs with severe bronchomalacia had a prolonged low-flow expiratory phase (defined as the time from flow <25% of PEF to end expiration) on F-t plot (Figure 3). This could be quantified by calculating the ratio of the expiratory low-flow phase to the entire expiratory period (ExpLF/Te), which was significantly higher in dogs with severe bronchomalacia compared to control dogs (0.76 vs 0.05, *p* < .001) (Table 3), even after excluding data recorded by the system prior to the upgrade.

### Volume-time (V-t) plot

**Figure 4.**
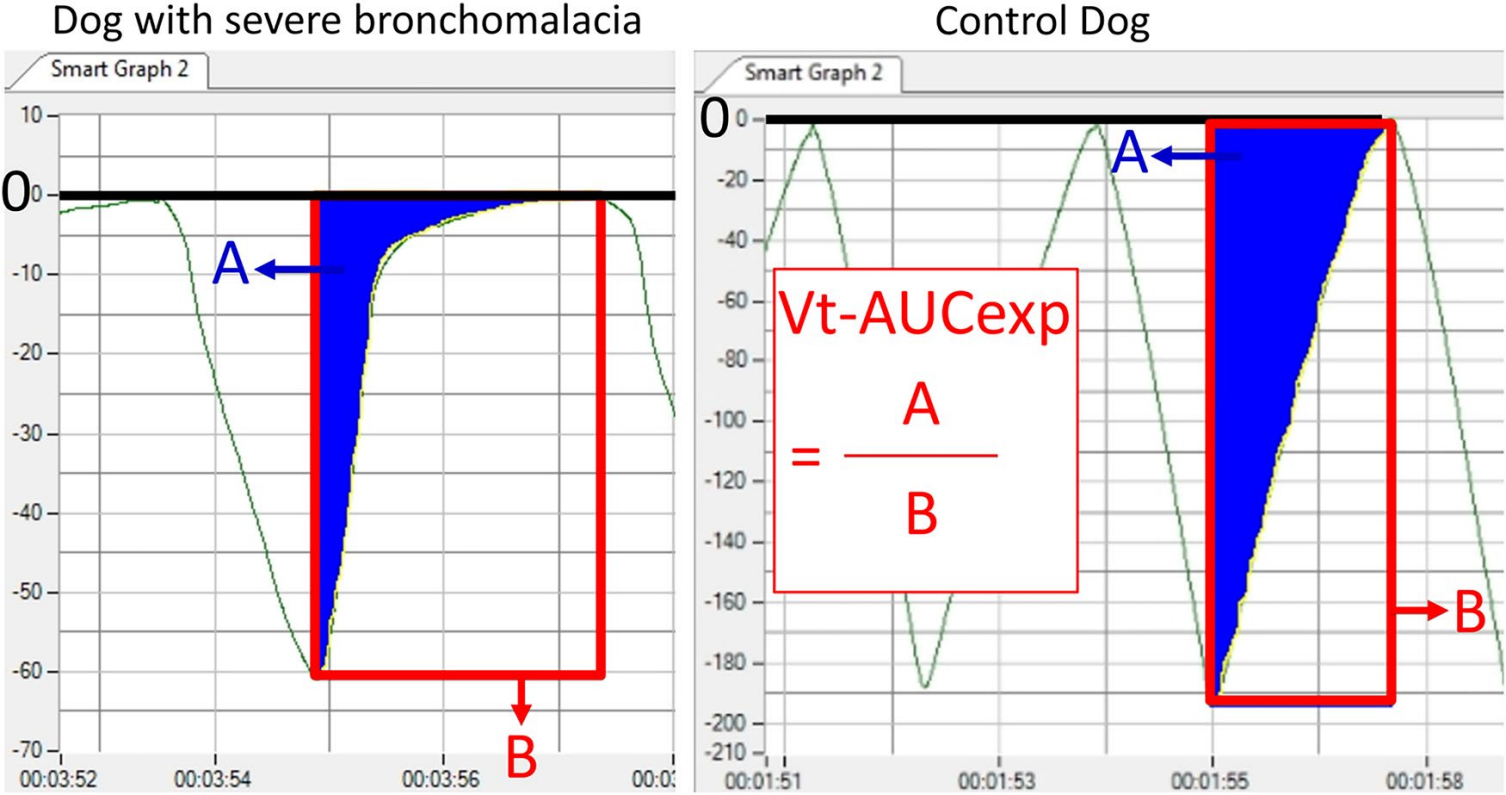
The volume-time (V-t) plots from a dog with severe bronchomalacia (left) and a control dog (right). an exponential shape of the expiratory curve was noted in dogs severe bronchomalacia, resulting in a low proportion of area under expiratory curve on V-t plot (Vt-AUCexp).

Dogs with severe bronchomalacia had an exponential shape of the expiratory curve on V-t plots (Figure 4). This feature could be quantified by calculating the proportion of the area under the expiratory curve on V-t plots (Vt-AUCexp), which was significantly lower in dogs with severe bronchomalacia compared to control dogs (19.3% vs 44.4%, *p* < .001) (Table 3), even after excluding data from the system prior to the upgrade.

### Sensitivity and specificity of spirometric parameters for predicting severe bronchomalacia

The receiver operating characteristic curves showed that the spirometric parameters ExpLF/Te and Vt-AUCexp had the highest AUC of 1.00 (95% confidence interval 0.88 to 1.00, *p* < .001). The cut-off value for ExpLF/Te of >0.14 had a sensitivity of 100% and a specificity of 100%. A cut-off for Vt-AUCexp of ≤31% had a sensitivity of 100% and a specificity of 100%.

## Discussion

The present study reports the ventilatory features of a novel functional phenotype in dogs with severe bronchomalacia. Lower respiratory tract obstruction has been recognized on F-V loops as either a concave or flat expiratory curve, produced by the development of expiratory flow limitation in a region with minimal area and transmural pressure within the airway after reaching the maximal flow (Campbell and Faulks 1965; Amis and Kurpershoek 1986; Rozanski and Hoffman 1999; Murgu and Colt 2006). Expiratory concavity is seen in cases with small airway obstructive disease (e.g. chronic obstructive pulmonary disease, asthma or bronchitis) (Miller and Hyatt 1973; Amis and Kurpershoek 1986; Padrid et al. 1990; Lin et al. 2014; Varga et al. 2016), whereas a flattened expiratory curve is shown in cases with intrathoracic large airway collapse or feline lower airway disease (Hyatt 1975; McKiernan et al. 1993; Weiner et al. 2003). In our study curves were neither concave nor flat, implying that ventilatory mechanics in these dogs with severe bronchomalacia are different from those reported in other airway diseases.

Although the term tracheobronchomalacia has long been used in veterinary medicine, the definition is disputed in the literature. Some clinicians use the term to refer to static collapse of large airways (Johnson and Pollard 2010; Bottero et al. 2013; Johnson et al. 2015), while others utilize bronchomalacia to describe dynamic collapse of smaller airways. Still others have attempted to create a classification scheme for documenting regions of static vs dynamic collapse (Bottero et al. 2013; Reinero and Masseau 2021). The term excessive dynamic airway collapse (EDAC) is also used in human medicine, representing airway lumen narrowing owing to excessive dynamic invagination of the membranous portion of the airway, which may result from hypotonia of myoelastic elements of the tracheobronchial tree (Murgu and Colt 2006; Represas-Represas et al. 2015; Heraganahally et al. 2020). While dynamic airway collapse is thought to be a component of tracheobronchomalacia, it has been proposed by some researchers in human medicine that tracheobronchomalacia and EDAC should be considered two separate entities, despite sharing some commonalities such as central airway obstruction and similar signs (e.g. chronic cough and expiratory respiratory difficulty) (Murgu and Colt 2006; Represas-Represas et al. 2015). Tracheobronchomalacia and EDAC have been increasingly recognized in human patients, and pulmonary function profiles in affected individuals were variable, including an obstructive pattern, normal performance, or even restrictive ventilatory characteristics (Majid et al. 2013; Heraganahally et al. 2020). Although pulmonary function test should not be used to determine whether malacic airways are present or not, the variability of ventilatory profiles in this group of human patients emphasizes the complexity of aerodynamics in dynamic airway collapse. The debate about tracheobronchomalacia and EDAC has not yet reached consensus in human medicine field (Murgu and Colt 2006; Represas-Represas et al. 2015; Heraganahally et al. 2020), but the essence of canine bronchomalacia is very likely to resemble the nature of these 2 disease entities.

The prolonged low-flow expiratory phase on the F-t plot in clinically affected dogs with severe bronchomalacia indicates expiratory flow limitation in the later phase of expiration. In normal tidal breathing, expiratory flow after peak expiratory flow should gradually and smoothly decrease to zero on the F-t plot (Clarke et al. 1994; Morris et al. 1998). The observation of a sudden fall in expiratory flow or the presence of very low flow for the remainder of exhalation after PEF has also been noted in some human patients with tracheobronchial collapse (Campbell and Faulks 1965; Majid et al. 2013). Hence this pattern on the F-t plot in dogs with severe bronchomalacia was not unexpected.

On a V-t plot, an exponential shape of the expiratory curve is a normal finding in a person performing forced expiration (Campbell and Faulks 1965; Kozlowska and Aurora 2005). We also observed this exponential shape of curves during expiration in dogs with severe bronchomalacia examined here, however, it is impossible to obtain a forced maneuver in dogs, and all spirometric signals in the current study were recorded under natural tidal breathing. Therefore, this finding in dogs with severe bronchomalacia is likely to correspond to abnormal abdominal effort in the expiratory phase. Future studies will evaluate the ability of this finding to quantify abnormal expiratory effort in dogs with bronchomalacia and will assess the impact of treatment on the exponential changes in volume.

Displays of flow versus volume illustrate additional information on aerodynamics. The relationship between flow and volume changes in our dogs indicates that the majority of volume was exhaled in the relatively early phase of expiration. This phenomenon is in contrast to the general concept of lower airway obstruction, which usually reveals low flow after expiring a small volume (leading to ‘concave’ expiratory curves) on F-V loops (Campbell and Faulks 1965; Murgu and Colt 2006). Prolonged expiration with a low flow rate on a F-t plot is a feature suggesting an obstructive disease process, whereas a large volume exhaled in the early phase of expiration implies a restrictive pattern of breathing (Majid et al. 2013; Heraganahally et al. 2020). This paradoxical finding in our dogs with severe bronchomalacia raises the suspicion that an abnormal restrictive physiology was demonstrated in these dogs. Interestingly, although an obstructive pattern was present in a majority of human patients with tracheobronchomalacia or EDAC, approximately 18% and 50% of patients, respectively, were found to have restrictive ventilatory defect in 2 studies (Majid et al. 2013; Heraganahally et al. 2020).

Another perspective that could indirectly support restrictive ventilatory characteristics in our dogs with severe bronchomalacia is the absence of global lung hyperinflation (increased space between the diaphragm and the heart with flattened diaphragm) (Gadbois et al. 2009) on thoracic radiography. Lung hyperinflation is a sign for air trapping that can be found in human or veterinary cases with asthma, bronchiolitis, or emphysema (Cooper et al. 2003; Winters et al. 2006; Gadbois et al. 2009). However, none of our dogs showed this typical image associated with lower airway obstruction despite having profound bronchomalacia. Moreover, global lung hyperinflation has not been described in any dog with bronchomalacia in previous studies (Johnson and Pollard 2010; Adamama-Moraitou et al. 2012; Singh et al. 2012; Bottero et al. 2013; Johnson et al. 2015; Hara et al. 2019). Therefore, ventilatory mechanics in canine bronchomalacia do not seem to be a simple obstructive process, and it is possible that a restrictive effect develops with time or with worsening severity of EDAC. It has been proposed that collapse of more central airways may not necessarily cause airflow obstruction, and expiratory flow limitation mainly results from collapse of more peripheral and smaller airways (Majid et al. 2013). One possible explanation for the ventilatory pattern observed in our dogs is a mixed obstructive and restrictive effect. Diffuse collapse of large bronchi could generate a restriction-like effect that prevents effective delivery of a large volume of air to the deeper airways, and an obstructive phase develops at late expiration as a result of the small volume of air reaching distal airways and alveoli.

While canine bronchomalacia and human tracheobronchomalacia/EDAC may share some commonalities in the concept of dynamic airway collapse, the clinical context in many aspects are different between dogs and human beings. Cases of isolated bronchomalacia without tracheal involvement are relatively rare in human medicine (Carden et al. 2005; Wallis et al. 2019), but quite common in dogs (Johnson and Pollard 2010). Chronic airway inflammation has been described as a risk factor for tracheobronchomalacia in humans (Murgu and Colt 2006; Majid et al. 2013), whereas the role of inflammation in canine bronchomalacia remains unclear (Johnson and Pollard 2010; Adamama-Moraitou et al. 2012; Bottero et al. 2013). Secondly, pulmonary function testing is routinely performed by forced expiratory maneuvers in human adults (Campbell and Faulks 1965), whereas ventilatory function in dogs is primarily evaluated under tidal breathing. Thus ventilatory parameters and the definition of an obstructive or restrictive pattern cannot be directly compared between the 2 species. For instance, while low PEF under ‘forced expiration’ was the most common finding on F-V loops in human patients with tracheobronchomalacia because of maximal flow limitation (Campbell and Faulks 1965; Majid et al. 2013), the PEF under ‘tidal breathing’ in dogs with severe bronchomalacia was higher than in normal control dogs due to the increased breathing effort (but far less than maximal expiratory capacity). Limitations for interpretation of spirometric values in veterinary patients should be considered, and methodologies specific for species or diseases should be developed and adopted.

Except for the differences in species, it remains unclear whether the size or breed of the dog would influence the observed ventilatory effect in this study. While many dogs in our study belong to small breeds and exhibit well-known tracheobronchomalacia (Johnson and Pollard 2010; Singh et al. 2012), it cannot be concluded if certain breeds or smaller dogs are more prone to this tidal breathing phenotype. In the future, investigating tidal breathing characteristics in a cohort of large-breed dogs showing expiratory effort due to severe bronchomalacia could help address these uncertainties. Furthermore, it is unknown what percentage of dogs with bronchomalacia will demonstrate this tidal breathing pattern without an extensive survey. The association between the presence of this functional phenotype and prognosis is also unclear at this moment. It could be challenging to generalize the findings to the entire canine population, and there are many unresolved questions to be answered.

This study had several limitations. First, two spirometric machines made by the same manufacturer were used in our study because the system was upgraded during the study period. The inconsistency of equipment could have caused some deviation of data, however equipment was calibrated as directed for the two different systems and the same analytic results were obtained after excluding data recorded with the old system. In studies in the human medicine field, researchers believed that some observation could be caused by mechanical artifacts due to different spirometer models (Murgu and Colt 2006). Given that the same ventilatory pattern was observed in dogs with severe bronchomalacia when using two different machines, it is more likely that the functional phenotype reported in this study was not an artifact. Second, it should be kept in mind during the interpretation of spirometric results that other disease states, such as concurrent tracheal collapse, may impact the spirometric results. Nevertheless, the extent of tracheal collapse observed in this study was significantly milder than that of bronchomalacia. Consequently, the influence on spirometric results was anticipated to be limited, given that the majority of study dogs presented with mild tracheal collapse (grade I and II), and none exhibited grade IV tracheal collapse. Finally, despite the absence of clinical suspicion of interstitial lung disease in the study dogs (all dogs had variable expiratory effort and none had a restrictive breathing pattern typical of pulmonary fibrosis), computed tomography and histopathology were not conducted to definitely exclude the possibility of underlying interstitial pathology. Therefore, it is unknown whether the current observation of mixed restrictive ventilatory characteristics is indicative of worsening bronchomalacia or an underlying interstitial disease process concurrent with bronchomalacia. Further evaluation such as high-resolution computed tomography, lung biopsy, or post-mortem necropsy should be considered in such clinical cases to clarify this issue in the future.

In conclusion, a tidal breathing phenotype characterized by an exponential shape of the expiratory curve on volume-time plots and prolonged low-flow expiratory phase on flow-time plots was identified in dogs with severe bronchomalacia. Among lower respiratory tract diseases, dogs with severe bronchomalacia show different ventilatory mechanics compared to simple inflammatory airway disease such as bronchitis in dogs or people or asthma in cats. The functional impairment in canine bronchomalacia seems to be not only an obstructive process, but also a restrictive ventilatory defect in some cases. The clinical application of tidal breathing patterns in disease progression, therapeutic response, or prognostic prediction in different functional phenotypes of canine bronchomalacia deserves further investigations.

## Supplementary Material

Supplemental Material
