# Supplementary material for: Quantifiable features of a tidal breathing phenotype in dogs with severe bronchomalacia diagnosed by bronchoscopy
Source: Vet Q. 2023 Sep 4;43(1):1–10. doi: 10.1080/01652176.2023.2252518 (PMC10478619; doi:10.1080/01652176.2023.2252518)

Supplementary Figure.

**Supplementary Figure 1**: The Receiver operating characteristic curves showed that the spirometric parameters ExpLF/Te and Vt-AUCexp had AUC of 1.00 (95% confidence interval 0.88 to 1.00, *P* < .001)


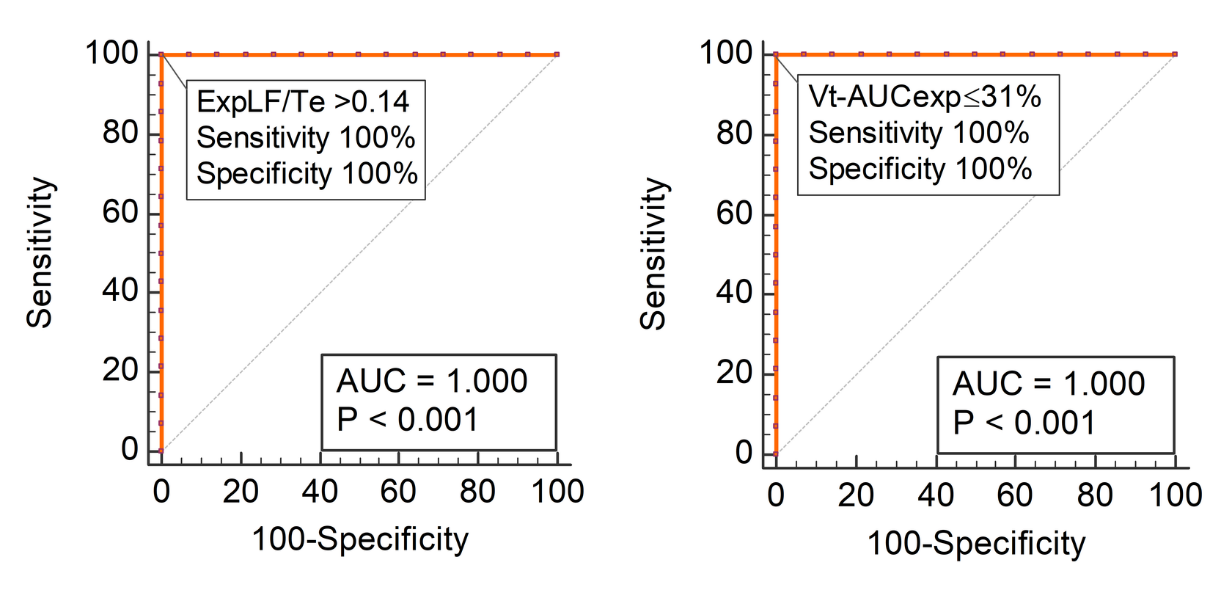

Supplement: Supplemental Material [file TVEQ_A_2252518_SM1785.docx]
